# Oriented cell division shapes carnivorous pitcher leaves of *Sarracenia purpurea*

**DOI:** 10.1038/ncomms7450

**Published:** 2015-03-16

**Authors:** Kenji Fukushima, Hironori Fujita, Takahiro Yamaguchi, Masayoshi Kawaguchi, Hirokazu Tsukaya, Mitsuyasu Hasebe

**Affiliations:** 1 1Department of Basic Biology, School of Life Science, SOKENDAI (The Graduate University for Advanced Studies), Okazaki 444-8585, Japan; 2 National Institute for Basic Biology, Myodaiji-cho, Nishigonaka 38, Okazaki, Aichi 444-8585, Japan; 3 Graduate School of Science, University of Tokyo, Hongo, Bunkyo-ku, Tokyo 113-0033, Japan

## Abstract

Complex morphology is an evolutionary outcome of phenotypic diversification. In some carnivorous plants, the ancestral planar leaf has been modified to form a pitcher shape. However, how leaf development was altered during evolution remains unknown. Here we show that the pitcher leaves of *Sarracenia purpurea* develop through cell division patterns of adaxial tissues that are distinct from those in bifacial and peltate leaves, subsequent to standard expression of adaxial and abaxial marker genes. Differences in the orientation of cell divisions in the adaxial domain cause bifacial growth in the distal region and adaxial ridge protrusion in the middle region. These different growth patterns establish pitcher morphology. A computer simulation suggests that the cell division plane is critical for the pitcher morphogenesis. Our results imply that tissue-specific changes in the orientation of cell division underlie the development of a morphologically complex leaf.

## Abstract (web summary)

The pitcher-shaped leaf of the carnivorous plant *Sarracenia purpurea* acts as a pitfall trap to capture small animals. Here, Fukushima *et al*. analyse pitcher leaf development and propose that this unusual shape evolved from ancestral planar leaves through changes in the orientation of cell division.

## Graphical abstract

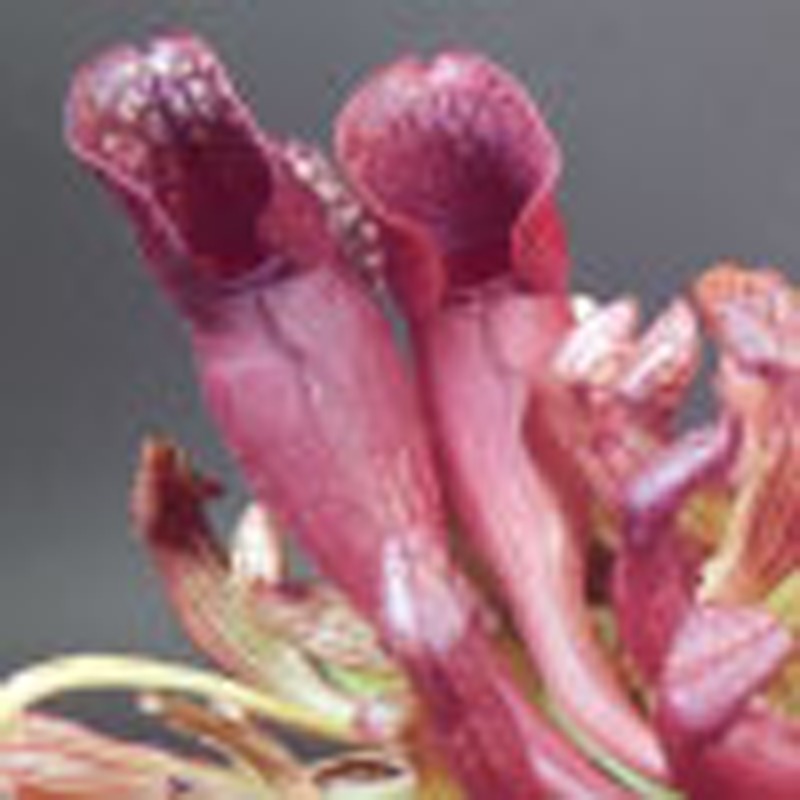

The emergence of novel morphology usually involves modifications of preexisting developmental programmes, but its basic mechanisms remain unclear, in particular for some drastic changes, such as the evolution of pitcher-shaped leaves in carnivorous plants. Leaves are usually planar, to allow efficient photosynthesis, but species in the family Sarraceniaceae produce pitcher-shaped leaves that function as pitfall traps to capture small animals. In planar leaf development, polarized primordia form a bifacial structure composed of adaxial and abaxial domains. Tissue closer to the shoot apical meristem becomes the adaxial domain and tissue farther from the meristem becomes the abaxial domain^1^. Adaxial and abaxial tissues have characteristic anatomical features, including vascular polarity and distinct patterns of expression of genes involved in leaf polarity. Several transcription factors involved in adaxial–abaxial development have been identified in *Arabidopsis thaliana*. For example, the class III homeodomain-leucine zipper transcription factor *PHABULOSA* (*PHB*) and the YABBY-type transcription factor *FILAMENTOUS FLOWER* (*FIL*) are expressed on the adaxial and abaxial sides, respectively^2^,^3^,^4^,^5^,^6^. Outgrowth of a leaf blade is induced at the boundary of adaxial and abaxial tissues; therefore, the final leaf shape depends on the position of the adaxial–abaxial boundary^7^.

Changes in adaxial–abaxial patterning contribute to the diversification of leaf morphology^7^,^8^. In a primordium of a conventional bifacial leaf^9^, such as that of *A. thaliana*, the complementary expression patterns of *PHB* and *FIL* are maintained from the tip to the base^3^,^4^,^5^,^10^ and blade outgrowth initiates at their expression boundary in the primordium, to form a flat structure (Supplementary Fig. 1). By contrast, *Tropaeolum majus* produces peltate leaves, which have a unifacial petiole attached to the central part of a bifacial leaf blade rather than at the margin^11^. The abaxial *FIL* expression pattern in the primordium of a peltate leaf is initially indistinguishable from that of a conventional bifacial leaf, but later *FIL* is expressed on both adaxial and abaxial sides of the primordium, in the proximal region where the unifacial petiole develops, leaving a bifacial structure in the distal region where the lamina forms^11^ (Supplementary Fig. 1). In addition, a mutation that attenuates the expression of adaxial determinants, including *PHB*, converts the conventional bifacial leaves of *A. thaliana* to peltate leaves^7^,^12^,^13^. Thus, the establishment of peltate leaves is related to changes in the expression patterns of the polarity genes^11^,^14^. Early studies showed that the outer morphology of young primordia in pitcher leaves of *Darlingtonia californica* resembles that in peltate leaves^15^,^16^, suggesting that peltate and pitcher leaves share a common developmental mechanism^9^,^15^. However, the development of the early primordia and the genes involved in polarity formation have not been examined.

In this study, we analysed leaf development in the purple pitcher plant *S. purpurea*. We first examined the spatiotemporal expression of *FIL* and *PHB* orthologues to test the hypothesis that pitcher leaves and peltate leaves develop by similar mechanisms. However, unlike peltate leaf primordia, pitcher leaf primordia did not show the prevailed *FIL* expression. We then examined the cell division pattern during pitcher development and found that oriented cell divisions in the adaxial tissue form a novel morphogenetic pattern that is distinct from that of both conventional bifacial and peltate leaves. A computer simulation showed that site-specific changes in the cell division plane could explain the novel morphogenetic pattern of the pitcher leaf. Taken together, our results show that local changes in the orientation of cell division lead to the novel morphology of pitcher leaves.

## Results

### Development of pitcher leaves

**Figure 1:**
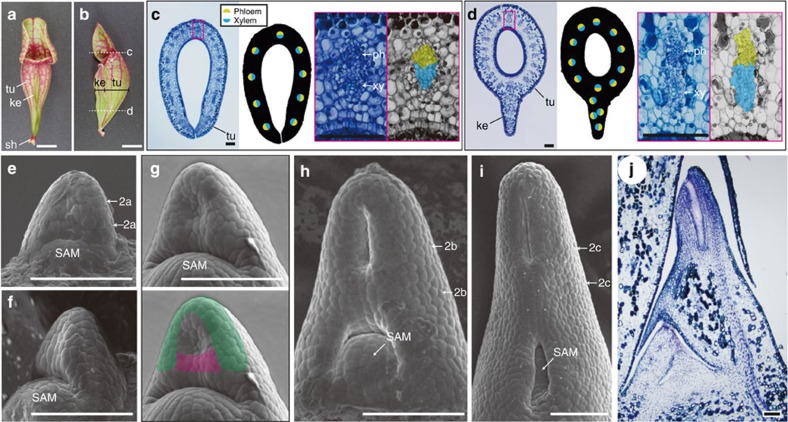
Morphology of *S. purpurea* pitcher leaves. (**a**,**b**) External morphology of mature pitchers: adaxial view (**a**) and lateral dissected view (**b**). tu, tube; ke, keel; sh, sheath. Dissected planes corresponding to those in **c** and **d** are indicated. (**c**,**d**) Transverse sections of immature pitchers of *ca*. 20 mm in length, stained with toluidine blue (left). Schematics of vascular polarity (middle) and magnified views of vascular bundles (right) are indicated. Vascular polarity is shown by the positions of adaxial element xylem (blue) and abaxial element phloem (yellow). ph, phloem; xy, xylem. (**e–i**) Scanning electron micrographs of developing pitcher primordia of *ca*. 70 μm (**e**,**f**), 100 μm (**g**), 200 μm (**h**) and 400 μm (**i**) in length. Adaxial (**e**) and lateral (**f**) views of *ca*. 70-μm primordia are shown. The leaf margin and adaxial ridge are shown in green and pink, respectively, in the lower panel of **g**. Approximate positions of sections in Fig. 2a–c are indicated by arrows in **e**, **h** and **i**, respectively. SAM, shoot apical meristem. (**j**) A longitudinal section of a pitcher primordium of *ca*. 1 mm in length. The scanning electron micrographs and toluidine blue-stained sections represent three to ten leaf primordia. Scale bars, 100 μm.

Mature pitcher leaves of *S. purpurea* are mainly composed of a tube, a keel and a sheath (Fig. 1a,b). In the tube, phloem bundles point towards the outer surface and xylem points towards the inner surface (Fig. 1c,d), indicating that this structure is bifacial, similar to the blades of conventional, planar leaves. In the keel, phloem bundles point towards the outer surface but xylem vessels face each other (Fig. 1d), indicating that the keel forms a distinct structure from the bifacial tube. We investigated the early development of *S. purpurea* pitcher leaves, using scanning electron microscopy. The adaxial surface of the incipient leaf primordium is flat (Fig. 1e,f), similar to that in conventional bifacial leaves^3^,^17^,^18^. When a primordium becomes ~100 μm long, an adaxial ridge connecting both sides of a leaf margin appears in the middle of the primordium (Fig. 1g), which is similar to the ‘cross zone’ protrusions in peltate leaves of *T. majus*^11^ and pitcher leaves of *D. californica*^15^. In *S. purpurea*, the adaxial ridge develops into a keel (Fig. 1a,b). When the primordium reaches ~200 μm in length, it becomes obvious that the proximal and distal parts of the adaxial ridge will form a keel and the adaxial side of the tube, respectively (Fig. 1h). As a result of growth in the leaf margin and the adaxial ridge, a hollow structure develops in the distal part of the primordium (Fig. 1i) and the continued growth of these regions deepens the hollow to form a pitcher shape (Fig. 1j).

### Polarity gene expression does not predict pitcher morphology

**Figure 2:**
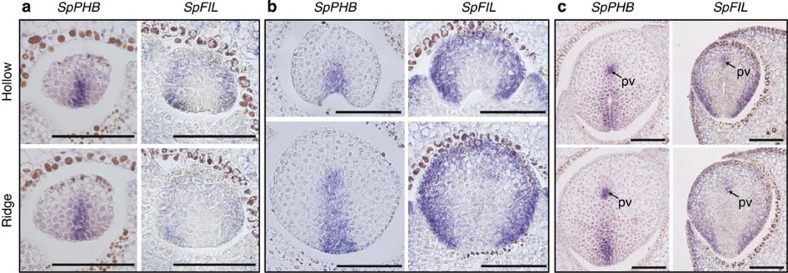
Expression patterns of *SpPHB* and *SpFIL* during pitcher development. (**a**–**c**) RNA *in situ* hybridization of *SpPHB* and *SpFIL* in pitcher primordia. Sections of primordia of *ca*. 70 μm (all sections in **a**), 160 μm (all sections in **b**), 370 μm (left sections in **c**) and 310 μm (right sections in **c**) in length. Sections are oriented with the abaxial side up and the adaxial side down. pv, provascular cells. Scale bars, 100 μm.

*PHB* and *FIL* transcription factors are expressed in the adaxial and abaxial surfaces, respectively, of some eudicot leaves^2^,^4^,^5^,^19^,^20^. *KANADI* is expressed in abaxial domains of both eudicots and monocots (reviewed in ref. 8), but transcripts of its orthologue was not detected in *S. purpurea* by RNA *in situ* hybridization. We isolated *PHB* and *FIL* orthologues in *S. purpurea* (*SpPHB* and *SpFIL*; Supplementary Fig. 2) and analysed their messenger RNA expression patterns during pitcher development (Fig. 2). Hybridization signals were not detected in sense probe experiments (Supplementary Fig. 3). In *S. purpurea*, the shape of each primordium varies slightly, probably because they are densely packed within the meristem. We therefore classified leaf primordia of similar heights as being in the same developmental stage. At least three primordia were examined for each developmental stage in RNA *in situ* hybridization (Fig. 2). In primordia that are ~70 μm long, *SpPHB* and *SpFIL* are expressed in the adaxial and abaxial surfaces of the incipient leaf primordia, respectively (Fig. 2a). We could not detect any difference in expression patterns between the distal and middle parts of primordia of this length. These expression patterns are similar to those in conventional bifacial leaves of *A. thaliana*^3^,^4^,^10^,^18^, although the relative sizes of the two expression domains may differ between *S. purpurea* and *A. thaliana*. In primordia that were ~100 μm in height, we detected *SpPHB* expression in the inner side of the hollow and in approximately six cell lines in the adaxial side of the ridge (Fig. 2b,c). Both signals were also detected in provascular cells. Unlike the peltate primordia of *T. majus*, *SpFIL* expression did not extend into the adaxial ridge (Fig. 2b,c), suggesting that pitcher and peltate leaves use different developmental mechanisms.

### Oriented cell division leads to differential morphology

**Figure 3:**
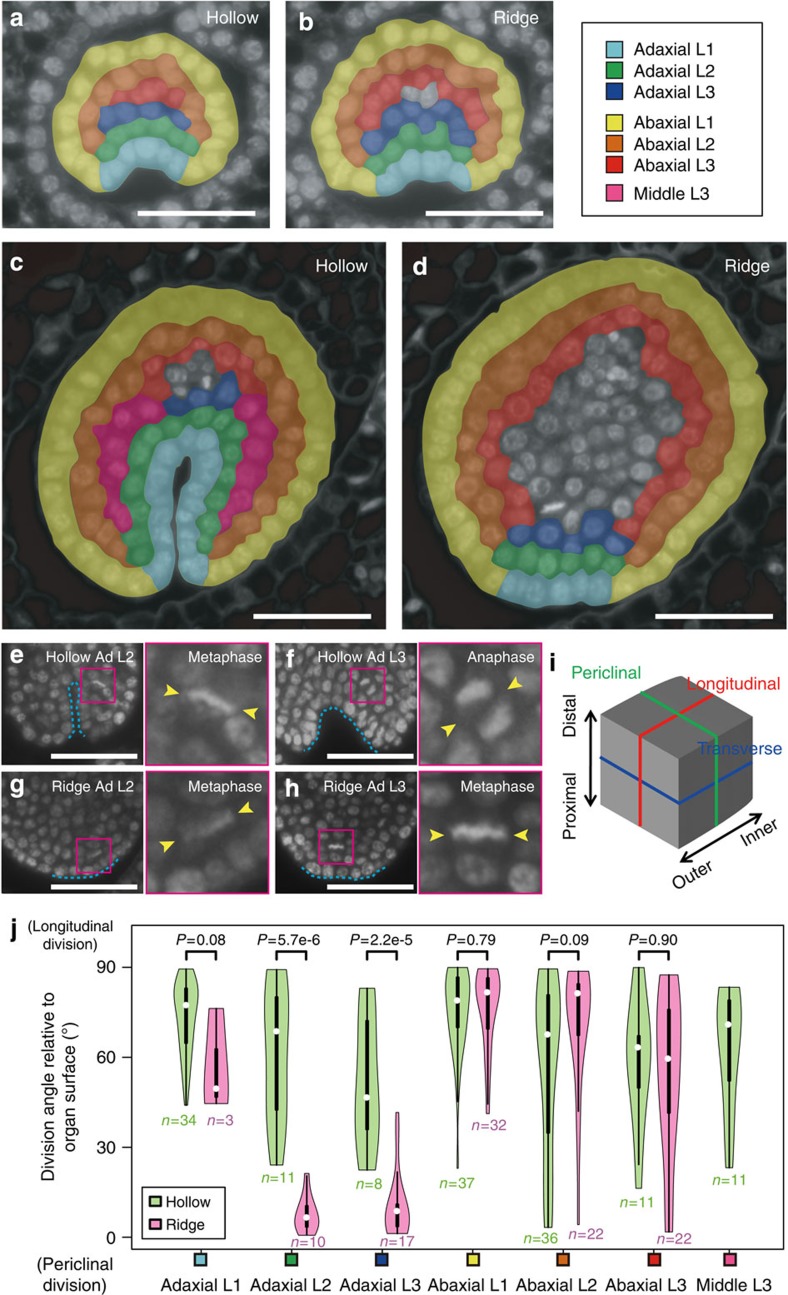
Oriented cell divisions in the hollow and ridge regions. (**a**–**d**) Classification of cell positions in transverse sections. Adaxial and abaxial layers 1 to 3 (L1 to L3) and middle layer 3 are indicated with different colours in sections of primordia of ~70 (**a**,**b**) and 250 (**c**,**d**) μm in height. (**e–h**) Examples of mitotic cells: longitudinal divisions in adaxial L2 (**e**) and adaxial L3 (**f**) cells of the hollow region and periclinal divisions in adaxial L2 (**g**) and adaxial L3 (**h**) cells of the ridge region. Dashed lines denote adaxial L1 cells. A magnified view of dividing cells are shown as an inset (magenta). Arrowheads indicate the spindle equator of dividing cells. (**i**) Definition of cell division planes. (**j**) Polarity of cell division orientation in transverse sections. In total, 254 metaphase cells from a total of 2,022 transverse sections prepared from 67 leaf primordia between 40 and 560 μm in height were measured. Division angles are illustrated as a violin plot. White circles indicate the median. Thick and thin lines cover ±1 and ±1.5 interquantile ranges, respectively. The vertical curve is an estimator of the density. Values close to 0° indicate periclinal division, whereas those close to 90° indicate longitudinal division. Cell division polarity in the hollow (green) and ridge (pink) regions was compared by the Mann–Whitney *U*-test. Scale bars, 100 μm.

In pitcher leaf primordia, the distal and middle regions develop a hollow and a ridge, respectively. Expression patterns of *SpPHB* and *SpFIL* were indistinguishable between the two regions in incipient flat primordia (Fig. 2a). These observations suggest that *SpPHB* and *SpFIL* expressions are not directly related to the morphological differentiation between the hollow and ridge. Plant development depends on the regulation of cell division planes because of the immobile nature of plant cells^21^. Therefore, we next examined the orientations of cell divisions in these two regions. Cell layers of each region were classified into layer 1–3 (L1–L3) from the outermost to innermost layers, in transverse sections (Fig. 3a–d). We defined the *SpPHB* expression domain as the putative adaxial domain. In the hollow region, L1 cells of the inner side of the developing hollow and adjacent L2 and L3 cells were defined as adaxial cells (Fig. 3a,c). In the ridge region, six adaxial cell files were defined as the adaxial domain (Fig. 3b,d), as *SpPHB* was expressed in approximately six epidermal cells (6.17±1.66 cells, mean±s.d.). The remaining cells were defined as abaxial cells, which presumably correspond to the *SpFIL* expression domain (Fig. 3a–d). In a primordium composed of five layers, the central layer was named the middle L3 layer. To measure the orientation of division, we identified M-phase cells in transverse sections stained with DAPI (4′,6-diamidino-2-phenylindole; Fig. 3e–h). We measured the angle between the tissue surface and the spindle equator (Supplementary Fig. 4), which was confirmed to correspond to the cell division plane (see Methods). Longitudinal divisions, which increase the number of cells in a cell layer, predominated in cells, except adaxial L2 and L3 cells in the ridge region, in which division planes were periclinal (Fig. 3i,j and Supplementary Table 1). These findings indicate that leaf tissues enlarge the area of a cell layer rather than the thickness, except in the adaxial L2 and L3 cells of the middle section, which undergo periclinal divisions to increase the number of cell layers, forming the ridge of the adaxial protrusion (Fig. 3d). The periclinal orientation was maintained until the primordia became at least 540 μm long (Supplementary Fig. 5). Simultaneously, the hollow region increased in area and the ridge region increased in thickness in the adaxial domain. The growth of the hollow region resembles the bifacial growth of conventional bifacial leaves, in which both adaxial and abaxial surfaces increase in area^1^. By contrast, the thickening growth of the ridge region observed in this study is characteristic to pitcher leaves and the two different growth modes together produce the pitcher shape.

As the mobile plant hormone auxin has been implicated in division plane regulation^22^, next we examined effects of auxin on pitcher development. Plantlets were grown on a medium containing a synthetic auxin 1-Naphthaleneacetic acid (NAA) or an auxin transport inhibitor 1-*N*-Naphthylphthalamic acid (NPA) for 4 weeks, and morphology of newly formed primordia was observed. Even in the presence of NAA or NPA in concentrations of 2–50 μM, leaves of *S. purpurea* clearly differentiated hollow and ridge regions (Supplementary Fig. 6). This result reduces the possibility of differential division plane regulation in hollow and ridge regions by auxin.

We also analysed how cell shape affects division plane in adaxial L2 and L3 cells. As a default mechanism, both animal and plant cells tend to make division plane at a right angle to cell long axis^23^,^24^. We measured geometry of dividing cells in transverse sections and compared contributions of cell shape-dependent ‘long-axis division rule’ and cell position-dependent periclinal/longitudinal orientation. Although division planes in both hollow and ridge regions were moderately correlated to periclinal/longitudinal orientation, almost no correlation was found between division plane and cell long axis (Supplementary Fig. 7). This indicates that cell position-dependent mechanisms mainly coordinate division plane orientation in adaxial L2 and L3 cells in both hollow and ridge regions.

### Computational modelling of pitcher leaf morphogenesis

**Figure 4:**
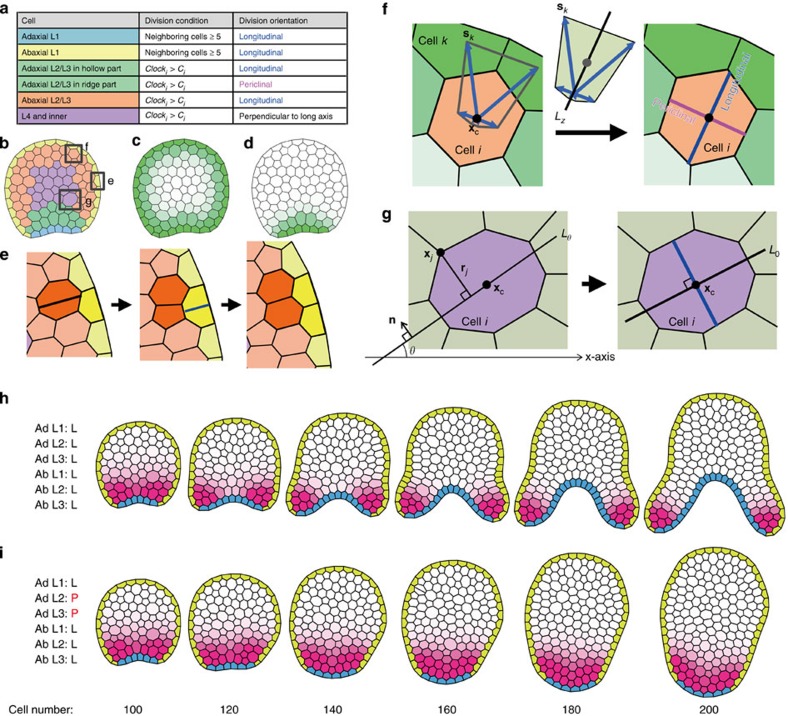
Computational simulation of cell proliferation during leaf development. (**a**) Summary of cell division condition and orientation according to cell position. (**b**) Example of classification of cell positions, which are distinguished by colours corresponding to those in **a**. Cells in squares are examples of L1, L2/L3 and inner cells, which are illustrated in **e**, **f** and **g**, respectively. Distribution of morphogens *v* (**c**) and *w* (**d**), with the darker shade representing a higher concentration. L1 cells divide longitudinally (blue line) in coordination with inner cell proliferation (**e**), L2/L3 cells divide longitudinally (blue line) or periclinally (magenta line) in response to the presence of a morphogen (green) (**f**) and more inner cells divide perpendicular to their long axis *L*_0_ (blue line) (**g**). (**h**) Cells in the outermost three layers are forced to divide longitudinally. (**i**) Adaxial L2 and L3 cells are forced to divide periclinally, whereas cells in other positions undergo longitudinal division. Simulations in **h** and **i** are performed with the same initial status. Adaxial and abaxial L1 cells are shaded blue and yellow, respectively. Magenta indicates the concentration of cell division-promoting morphogen, which diffuses from the boundary of adaxial and abaxial L1 cells. Darker shade represents higher concentration. Preset division planes of cell layers are indicated on the left side: L, longitudinal division; P, periclinal division.

To examine whether the different cell division patterns are sufficient to explain bifacial growth in the hollow region and protruding growth in the ridge, we developed a computational model called vertex dynamics model^25^,^26^ to simulate proliferating plant tissues (Fig. 4a–g, see Methods for detail). Plant organs develop by cell division and cell expansion (reviewed in ref. 27). In early leaf development, cells actively divide and cell sizes are relatively constant and small. Cell divisions later cease and cell expansion activity intensifies instead. As cell sizes were constant and its increase was undetectable during the developmental stage we analysed (Supplementary Fig. 8), we concluded that hollow and ridge differentiate within the cell division phase. Therefore, we constructed a computational model focusing on cell division in which cell expansion occurs after cell division only to maintain constant cell sizes. Transverse sections of plant tissues were modelled as a two-dimensional aggregate of polygonal cells drawn as connections of vertices. On cell division, a new vertex connection was introduced to divide one cell into two daughter cells. As we observed higher cell division activity in the marginal regions than in other regions (Supplementary Fig. 9), as found in *A. thaliana*^18^, we introduced a cell division-promoting morphogen that diffuses from the boundary of adaxial and abaxial epidermal cells (magenta in Fig. 4h,i). Leaf primordia before pitcher morphogenesis have a slight depression in the adaxial side (Fig. 3a,b) and this was found to be important for simulating leaf morphogenesis, because proper bifacial growth was suppressed when simulations were started from cell aggregates with round shape (Supplementary Fig. 10). Therefore, pitcher leaf morphogenesis was simulated using initial shape with depression in adaxial side. When starting with 100 cells and forcing L1, L2 and L3 cells of both adaxial and abaxial tissues to divide longitudinally as observed in the hollow region of actual leaf primordia (Fig. 3j), bifacial growth was recapitulated (Fig. 4h). When adaxial L2 and L3 cells were forced to divide periclinally (Fig. 3j), an adaxial protrusion formed (Fig. 4i).

To understand how cell division activity affects growth patterns, we changed three parameters related to division activity: the diffusion coefficient of the cell division-promoting morphogen, the synthesis rate of the morphogen and the division-inducing efficiency of the morphogen (Supplementary Fig. 11). Although simulated morphology fluctuates when the parameters are changed, the model stably reconstructed bifacial growth and adaxial protrusion. This indicates that the morphological differences between the two growth patterns result from differences in cell division orientation.

Together, these modelling results indicate that differences in the orientations of cell divisions in the adaxial tissues should be sufficient to produce the distinct growth patterns of bifacial growth and adaxial protrusion, which combine to form the pitcher morphology.

## Discussion

In the present study, we analysed pitcher leaf development in *S. purpurea* and found that the pitcher shape is established through differential cell division patterns between the hollow and ridge regions of a leaf primordium. The morphology of the pitcher primordium and the expression patterns of *PHB* and *FIL* orthologues before formation of the hollow are similar to those of conventional bifacial leaves and peltate leaves during the early developmental stages (Fig. 2a). Subsequently, pitcher morphology is established through differential cell division patterns in the leaf primordium (Fig. 3). In the hollow part of the leaf primordium, longitudinal cell divisions predominated in L1, L2 and L3 cells of both the adaxial and abaxial surfaces (Fig. 3), as in conventional bifacial leaves^1^. By contrast, in the ridge region, periclinal cell divisions predominated in the L2 and L3 cells of the adaxial surface (Fig. 3) and resulted in a protruding ridge that formed a keel (Fig. 1a,b). The different modes of growth between the hollow and ridge regions form a tube structure. Therefore, the spatial regulation of oriented cell divisions in the leaf primordium is key for pitcher formation.

We used computer simulation to examine the effect of growth parameters because of difficulty in experimental manipulation of multicellular dynamics *in planta*. In addition to cell division orientation resulting in the specific morphology of hollow and ridge regions (Fig. 4), initial morphology (Supplementary Fig. 10) and spatial distribution of cell division frequency (Supplementary Fig. 11) appear to contribute to leaf development. Changes in these two parameters result in attenuation of bifacial growth and this raises the possibility that proper initial morphology and cell division frequency are required for blade formation in hollow region of pitcher leaves as well as in a leaf blade of other types of leaves. This view is concordant with previously described phenotypes of *Arabidopsis* mutants defective in two *WUSCHEL-RELATED HOMEOBOX* (*WOX*) genes that have been proven to function in leaf blade formation^18^,^28^. Double mutants of *wox1* and *pressed flower* abolish higher cell division activity in leaf margins and show attenuated blade formation^18^, exhibiting patterns similar to simulated morphogenesis (Supplementary Fig. 11). Although division plane determines basic morphology of hollow and ridge regions, other parameters are likely to additionally function to form final shapes for proper leaf development. Further sophistication of the model may be beneficial for more detailed analyses because of the complexity of multicellular dynamics. Incorporation of the experimentally measured dynamics of cell shape and growth rate will allow more robust modelling. Furthermore, application of three-dimensional vertex modelling^29^,^30^ to pitcher morphogenesis may give additional insights into how distinct growth patterns are coordinated and integrated in a pitcher primordium.

The *PHB* and *FIL* expression patterns of incipient leaf primordia are conserved in conventional bifacial leaves of diverse flowering plants including *A. thaliana*^3^,^4^, *Antirrhinum majus*^19^ and *Cabomba caroliniana*^31^. The expression patterns in the early primordia are also conserved in leaves with varied morphology, including peltate leaves^11^. Together with our results on pitcher development, these observations show that polarized gene expression at the initial stage is evolutionarily conserved in different types of leaves. However, after the conserved stage of polarity establishment, organ-specific differences appear, including spatial expression changes of polarity markers in peltate leaves and cell division patterns without polarity marker expression changes in pitcher leaves.

Because of the partly similar developmental processes between peltate and pitcher leaves, pitcher leaves have been hypothesized to be a modified form of the peltate leaf^9^,^32^. We found that the pitcher leaf architecture is established through modified cell division patterns rather than a change of leaf polarity indicated by polarity marker gene expression, which underlies peltate leaf development^7^,^11^ (reviewed in ref. 8). Therefore, the developmental mechanisms of pitcher leaves appear to be distinct from those of peltate leaves. Although the mechanisms of division plane regulation remain largely unknown, both chemical and physical signals are implicated in this process. It is reported that auxin participates in division plane regulation in embryonic development^22^, although auxin addition did not disturb hollow and ridge formations (Supplementary Fig. 6). Given that auxin acts in a tissue-specific manner (for example, see ref. 33), visualization of endogenous auxin distribution in transgenic plants with auxin-responsive marker genes may give clearer results to test the hypothesis of auxin’s involvement of division plane regulation in pitcher development. Another candidate of division plane regulator is physical properties. Although our results showed that so-called ‘long-axis division rule’ does not act in the tissues we analysed (Supplementary Fig. 7), physical properties can orient division plane without affecting cell shapes by mechanical feedback, in which mechanical forces coordinate growth dynamics by modulating chemical signals^34^,^35^. Signalling molecules or physical properties that do not alter the expression of polarity markers might have been differentially regulated during the evolution of pitcher leaves.

Carnivorous plants evolved at least five times in flowering plants and pitcher morphology evolved three times^36^. However, the leaf morphology of sister taxa does not provide insight into the intermediate morphology of pitcher leaf evolution. In addition, fossil records are scarce. Therefore, the evolutionary processes and mechanisms underlying pitcher leaf formation have remained mostly unknown^32^. Major morphological changes appear to be due mainly to mutations with large effects or to the accumulation of many changes with small effects^37^. As cell walls limit the mobility of plant cells, cell division activity and direction usually have critical roles in shaping plant organs. Changes at the cellular level, such as the changes of oriented cell divisions found in this study, probably function as the source of major morphological changes at the organ level during the evolution of pitcher leaves and other distinctive organs.

## Methods

### Plant materials

*S. purpurea* ssp. *venosa* L. plantlets were purchased from Daisho-en Nursery (Numazu, Japan) and cultivated in a greenhouse. Axenically grown plants were obtained from CZ Plants Nursery (Trebovice, Czech Republic) and were subcultured on half-strength Murashige and Skoog plate medium^38^ supplemented with 3% sucrose, 1 × Gamborg’s vitamins, 0.1% 2-(N-morpholino)ethanesulfonic acid, 0.05% Plant Preservative Mixture (Plant Cell Technology) and 0.3% Phytagel, at 25 °C under continuous light. Voucher specimens were deposited in The Herbarium of the University of Tokyo (TI). Accession numbers are KF001 and KF002 for axenically grown and greenhouse-grown plants, respectively.

### RNA extraction

Fresh shoot apices of ~10 mm diameter were excised from soil-grown plants, washed with tap water and ground in liquid nitrogen, using a mortar and pestle. Total RNA was extracted using PureLink Plant RNA Reagent (Life Technologies).

### Cloning of *SpPHB* and *SpFIL*

Complementary DNA was synthesized from total RNA using SuperScript III Reverse Transcriptase (Life Technologies). Gene fragments were amplified with degenerate primers (Supplementary Table 2), cloned into pGEM-T Easy Vector (Promega) and sequenced with the ABI Prism 3100 Genetic Analyzer (Applied Biosystems). Next, 3′-terminal sequences were obtained by rapid amplification of cDNA ends using GeneRacer (Invitrogen) and gene-specific primers (Supplementary Table 2). Corresponding transcript sequences retrieved from RNA-seq data of HiSeq 2000 (Illumina) were also included (DNA Data Bank of Japan BioProject ID: PRJDB3436). DDBHJ accession numbers are as follows: *SpPHB*, AB938211; *SpFIL*, AB938212; and *SpHIS4*, AB938214.

### Phylogenetic analysis

Phylogenetic relationships among *SpPHB*, *SpFIL* and their homologues in the annotated genomes of *A. thaliana* (TAIR10; ref. 39), *Populus trichocarpa* (v3; ref. 40), *Mimulus guttatus* (v1.1, distributed by Department of Energy Joint Genome Institute at http://www.jgi.doe.gov/), *Solanum lycopersicum* (ITAG2.3; ref. 41), *Aquilegia coerulea* (distributed by Joint Genome Institute), *Oryza sativa* (MSU Release 7.0; ref. 42) and *Picea abies*^43^ were analysed. Coding sequence datasets were obtained from The Arabidopsis Information Resource (http://www.arabidopsis.org/), Phytozome v9.1 (http://www.phytozome.net/) and ConGenIE (http://congenie.org/). TBLASTX^44^ searches of *SpPHB* and *SpFIL* sequences were performed against the above coding sequence datasets with *E*-value cutoffs of 1*e*−160 and 1*e*−20, respectively. After sequence retrieval, multiple alignments were prepared with MAFFT 6.956 (ref. 45) and ambiguous codons were removed using trimAl^46^ with the ‘gappyout’ option implemented in Phylogears2-2.0.2013.03.15 (http://www.fifthdimension.jp/products/phylogears/). Phylogenetic trees were reconstructed by the maximum likelihood method using RAxML 7.5.3 (ref. 47) with the general time-reversible model of nucleotide substitution and four discrete γ-categories of rate heterogeneity (GTRGAMMA option). Support for nodes was estimated by rapid bootstrapping with 1,000 replications.

### Scanning electron microscopy

Shoot apices and leaves of axenically grown plants were excised with fine forceps, frozen in liquid nitrogen and immediately observed with a scanning electron microscope XL30 (FEI, Hillsboro, Oregon, USA), with an accelerating voltage of 10 kV.

### Serial section preparation and toluidine blue staining

Shoot apices of soil-grown plants were fixed overnight in 4% paraformaldehyde (pH 7.2). The fixative solution was sequentially replaced with a series of water–ethanol, ethanol–xylene and xylene–Paraplast Plus (Sigma-Aldrich), to prepare paraffin-embedded samples. Serial sections were made using a rotary microtome (Leica RM 2155, Leica) and were affixed to glass slides overnight at 42 °C. Next, 9-μm-thick serial sections were rehydrated through xylene–ethanol and ethanol–water series, and stained with 0.1% toluidine blue in 0.1 M phosphate buffer (pH 7.0). The preparations were mounted in Entellan New (Merck Millipore). Section images were taken using a digital camera (DP70, Olympus) coupled to a microscope (BX-51, Olympus).

### RNA *in situ* hybridization and signal quantification

Cloned gene fragments were amplified using the M13-21 and RV-17mer primers (Supplementary Table 2). Digoxigenin (DIG)-labelled antisense RNA probes were prepared using the DIG RNA Labeling Kit (Roche Applied Science). Next, 9-μm-thick serial sections were rehydrated, treated with 0.5 μg ml^−1^ Proteinase K for 30 min at 37 °C, re-fixed in 4% paraformaldehyde (pH 7.2) for 10 min, and then dehydrated in a water–ethanol series. RNA probe hybridization was performed overnight in a humid chamber at 50 °C. After the samples were washed twice with 4 × SSC buffer at 50 °C for 20 min, the slides were treated with 50 μg ml^−1^ RNase A at 37 °C for 60 min, washed twice in 0.5 × SSC at 50 °C for 20 min and then blocked with Blocking Reagent (Roche Applied Science). Signals were detected by incubating the samples in Anti-DIG-AP (Roche Applied Science) for 90 min and NBT/BCIP solution (Roche Applied Science) overnight. After brief dehydration in water–ethanol and ethanol–xylene series, the preparations were mounted in Entellan New (Merck Millipore). Section images were taken using a digital camera (DP71, Olympus). Using ImageJ 1.47k (National Institutes of Health, MD, USA), segmented lines were drawn through the centres of the epidermal cells and grey values were obtained for each pixel along these lines (Supplementary Fig. 12). Subsequently, a B-spline curve was drawn through the data points. The boundary of signal-positive and -negative cells was determined by calculating the local maximum and local minimum of backward difference of fitted values (Supplementary Fig. 12).

### Analyses of cell division orientation

To visualize spindle equators in inner tissues, 8-μm-thick serial sections were prepared from axenically grown plants. The prepared sections were rehydrated on slides, stained with 1 μg ml^−1^ DAPI in McIlvaine’s buffer (pH 7.0) and then mounted in 50% glycerol. Mitotic chromosomes were observed using the filter set WU (Olympus) on a microscope (BX51, Olympus). Although some plant tissues decouple the spindle equator from division plane by equator reorientation, the angle of spindle equators was not significantly different between metaphase and anaphase–telophase, at which point equator reorientation may occur^48^,^49^,^50^ (Supplementary Fig. 13). This indicates that the position of a spindle equator corresponds to that of a division plane in the pitcher leaves of *S. purpurea*.

### Hormone treatments

Axenically grown plants were transferred to the half-strength Murashige and Skoog medium containing 2, 10, or 50 μM of NAA or NPA, and were grown at 25 °C under continuous light for 4 weeks.

### Statistical analyses

All statistical analyses were performed using R 3.0.1. Comparisons were considered statistically significant when *P*-values were <0.05. Multiple comparisons were corrected with Bonferroni’s procedure. Angular data were processed with R package ‘circular’.

### Vertex model

Leaf primordium development was modelled to examine the effect of cell division orientation by coupling vertex dynamics and chemical dynamics. To simplify the model system, transverse sections of primordia, in which each cell is represented by a polygon specified by surrounding vertices, were considered. If it is assumed that vertices are embedded in a viscous medium and have no mass, the equation of motion for vertices is given by


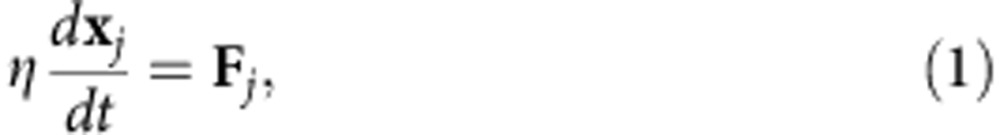


where **x**_*j*_ is the position vector of vertex *j*, **F**_*j*_ is the total force acting on vertex *j* and *η* is the viscosity coefficient. The acting force is often described using potential *U* as follows:


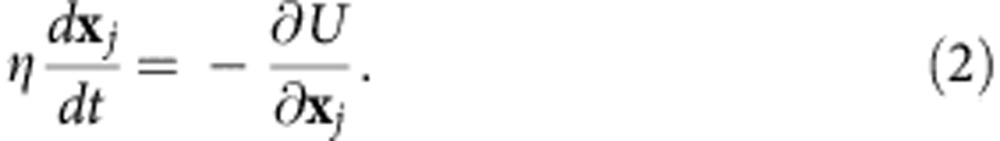


Thereby, vertices move so as to minimize the potential energy and we can describe various dynamics of vertices by changing potential energy function^25^,^51^,^52^. Vertex model has been extensively used for investigating cellular mechanisms of morphogenesis in animals (see references cited in ref. 52) and in plants^23^,^53^,^54^,^55^,^56^,^57^,^58^,^59^. In models used in these studies, acting force on the vertex is frequently described based on the elasticity of cell area and bond tension of cell edge. Accordingly, in our model, potential energy is given by the following simple form with some modifications:


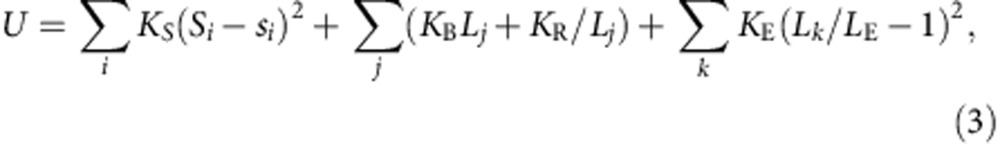


where *S*_*i*_ is the area of cell *i*, *L*_*j*_ is the length of edge *j*, *L*_*k*_ is the length of outermost edge *k*, and *K*_S_, *K*_B_, *K*_R_, *K*_E_ and *L*_E_ are constants. The first term of the right-hand side represents area elasticity, where cell *i* has a target area *s*_*i*_ that depends on its edge number *N*_*i*_ and is given by the relative area of *N*_*i*_-sided regular polygon to that of regular hexagon, namely, *s*_*i*_=*N*_*i*_ tan(*π*/6)/6 tan(*π*/*N*_*i*_). *K*_S_ denotes the elastic constant. The second term describes the conservation force of edge length, where each edge has the target length 
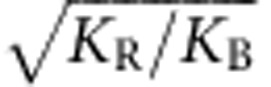
 at which the potential energy function reaches a minimum. This condition ensures that neighbouring vertices do not become extremely close because of repulsive force. The third term indicates the elastic force that maintains the outermost edges at a constant length *L*_E_ with the elastic coefficient *K*_E_, which reflects that the outer cell wall is usually thickened and stiffened by a cuticle.

In animal cell models, it is usually assumed that vertices are reconnected to mimic cell movement if an edge becomes extremely short^25^,^51^,^52^. In contrast, because the plant cell wall prevents changes in the relative position of cells, such reconnection process is not permitted in our model. In addition, neighbouring vertices are prohibited to be extremely close by potential energy as described above.

Programmes were written in C and the vertex position (**x**_*j*_) was iteratively calculated every time step Δ*t*=0.005, using the fourth-order Runge–Kutta method.

### Model of cell division in epidermal cells

In the epidermis, longitudinal divisions are observed in the adaxial domain where inner cells divide longitudinally, but rarely in the ridge part where inner cells divide periclinally (Fig. 3j). This evidence, together with a previous report on inter-cell-layer signalling^60^, suggests that epidermal cells divide in coordination with inner cell proliferation. In transverse sections of leaf primordia in *S*. *purpurea*, it is observed that most epidermal cells have less than five neighbouring cells (Supplementary Fig. 14). Accordingly, in our model, when an epidermal cell has five neighbouring cells (or six edges), it is longitudinally divided by a line through the midpoints of the outermost and innermost edges, and then returns to having four neighbouring cells (Fig. 4e, dark yellow cells). This division condition ensures that an epidermal cell proliferates, while keeping less than five neighbouring cells throughout primordial growth.

### Cell division clock

Epidermal cells are differentiated into *PHB*-positive and *FIL*-positive states, which correspond to adaxial and abaxial L1 cells shown in blue and yellow, respectively, in Fig. 4h,i. Cell division is active in the leaf margin (Supplementary Fig. 9), that is, near the epidermal boundary of the adaxial and abaxial domains. Thus, we introduced a morphogen that promotes cell division:


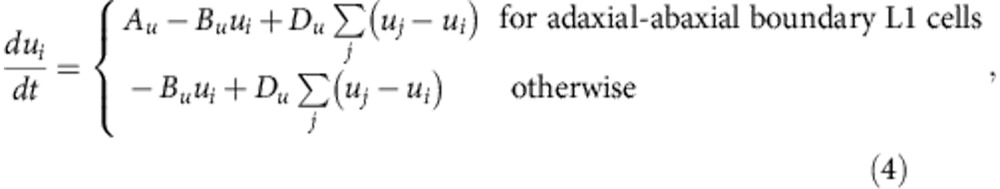


where *u*_*i*_ and *u*_*j*_ are the morphogen concentrations of cell *i* and its neighbouring cell *j*, respectively, and *A*_*u*_, *B*_*u*_ and *D*_*u*_ are the synthesis rate, degradation rate and diffusion coefficient, respectively. Morphogen *u* is synthesized in L1 cells of the adaxial–abaxial boundary, diffuses and decreases in concentration with distance from the boundary and stimulates cell division as described below (Fig. 4h,i, magenta).

In contrast to the epidermis, non-epidermal cells divide depending on their cell division clock, which is promoted by the morphogen (*u*_*i*_) and cell area (*S*_*i*_) as follows:


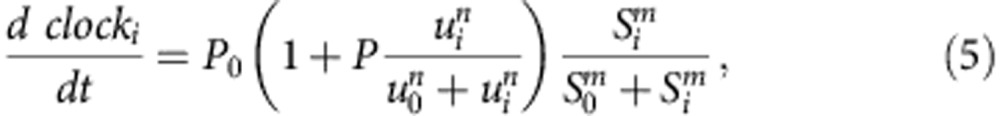


where *clock*_*i*_, *u*_*i*_ and *S*_*i*_ are the clock, morphogen concentration and area of cell *i*, respectively, and *P*_0_, *P*, *u*_0_, *S*_0_, *n* and *m* are constants. Cells divide if their clock exceeds a threshold (that is, *clock*_*i*_>*C*_*i*_) and then the clock is reset to zero in their daughter cells, where *C*_*i*_ is the threshold of cell *i* and is given by *C* with 10% fluctuation.

### Model of cell division orientation in inner cells

Cell division orientation is determined according to experimental observations (Fig. 3j). That is, cells divide longitudinally or periclinally in the outermost three cell layers (that is, L1, L2 and L3) (Fig. 4a). On the other hand, more inner cells than L3 cells divide perpendicular to their long cell axis. To determine the long axis of a dividing cell, the line *L*_*θ*_ that runs through the cell centre (**x**_c_) and forms angle *θ* with the *x* axis was considered (Fig. 4g). *L*_*θ*_ satisfies


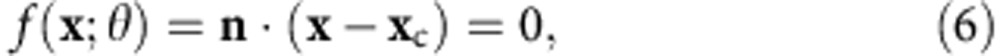


where **n**=(−sin*θ*, cos*θ*) is a normal vector of *L*_*θ*_, 
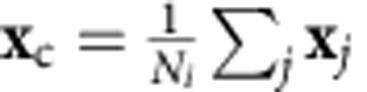
 and *N*_*i*_ is the centre position and vertex number of dividing cell *i*, respectively, and **x**_*j*_ is the position of vertex *j*. The sum of squared distances was then introduced:


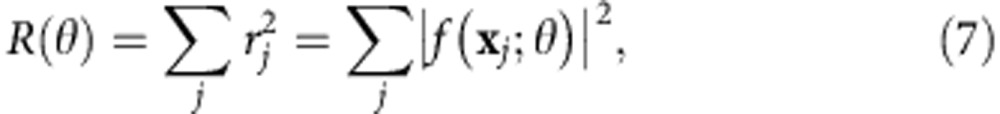


where *r*_*j*_ is the distance between vertex *j* and line *L*_*θ*_. *R*(*θ*) reaches a minimum at


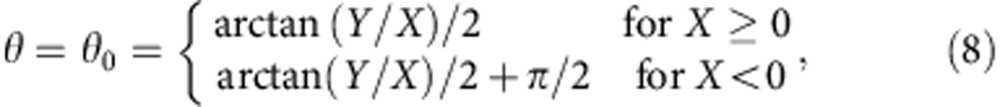


where 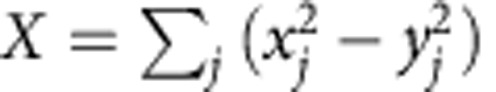, 
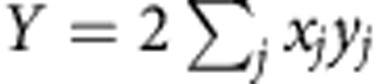
 and (*x*_*j*_, *y*_*j*_)=**x**_*j*_–**x**_c_. This indicates that the line *L*_0_ satisfying *f*(**x**,*θ*_0_)=0 corresponds to the long cell axis, and accordingly the focal cell is divided by the line that runs through the cell centre **x**_c_ and is perpendicular to *L*_0_ (Fig. 4g, blue line).

### Model of cell division orientation in L2 and L3 cells

In L2 and L3, cells divide longitudinally, with the exception of adaxial L2 and L3 cells of the ridge region, which divide periclinally (Fig. 3j). Adaxial L2 and L3 are defined as the L2 and L3 cells that are connected with adaxial L1 and L2, respectively. To determine the orientations of the longitudinal and periclinal divisions, assumed morphogens were introduced as follows:


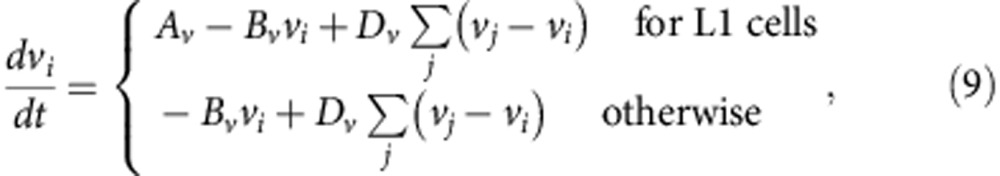



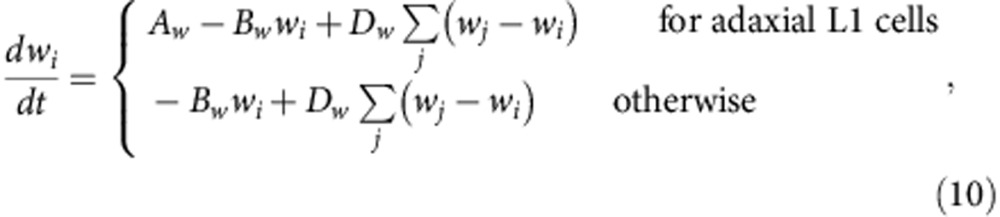


where the notations are the same as those in equation (4) after replacing *u* with *v* or *w*. Morphogens *v* and *w* are synthesized in the epidermis and adaxial epidermis, respectively, diffuse and decrease in concentration with distance from their synthesis region (Fig. 4c,d) and affect the orientations of longitudinal and periclinal cell divisions, respectively, in L2 and L3. Next, the following was introduced:


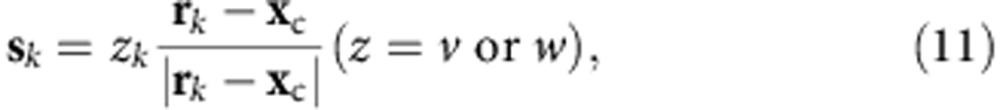


where ***x***_c_ is the centre position of the dividing cell *i*, *z*_*k*_ (*v*_*k*_ or *w*_*k*_) is the morphogen concentration of neighbouring cell *k* and **r**_*k*_ is the midpoint position of the edge shared with cell *i* and cell *k* (Fig. 4f). Vector **s**_*k*_ is oriented towards cell *k* with the same size as the morphogen concentration of cell *k* (that is, |**s**_*k*_|=*z*_*k*_). When the polygon consisting of the vertices with position vector **s**_*k*_ is considered, this polygon elongates towards higher concentrations of morphogen *z*, and thereby its long axis will be oriented longitudinally. This long axis can be determined by the same method as that described in the previous section and is denoted here as *L*_*z*_ by morphogen *z* (*v* or *w*). As a result, in L2 and L3, a cell is divided by the line that runs through the cell centre and is parallel to *L*_*v*_ in the case of longitudinal division (Fig. 4f, blue line), and by the line perpendicular to *L*_*w*_ in periclinal division (Fig. 4f, magenta line).

### Initial and parameter conditions

At an initial stage of leaf primordium development (70 μm in size), the transverse sections of the hollow and the ridge regions are similar to each other in shape and adaxial–abaxial patterning (Fig. 3a,b). Thus, we used this primordial stage as an initial condition for numerical simulations, in which primordial sections consist of ~100 cells and have a round shape with a small depression in the adaxial side. Initial adaxial L1 is given as a string of six epidermal cells at the depression and the other epidermal cells are defined as initial abaxial L1 (Fig. 4h,i, 100 cells). Parameter values used in Fig. 4h,i are as follows: *η*=1.0, *K*_S_=1.0, *K*_B_=0.1, *K*_R_=0.001, *K*_E_=0.005, *L*_E_=1.3, *A*_*u*_=2.0, *A*_*v*_=1.0, *A*_*w*_=1.0, *B*_*u*_=1.0, *B*_*v*_=1.0, *B*_*w*_=1.0, *D*_*u*_=1.0, *D*_*v*_=0.2, *D*_*w*_=0.2, *P*_0_=1.0, *P*=20.0, *u*_0_=0.03, *S*_0_=1.2, *n*=2, *m*=8 and *C*=10,000.

Programmes were written in C, and morphogen concentrations (*u*_*i*_, *v*_*i*_ and *w*_*i*_) and cell division clock (*clock*_*i*_) were iteratively calculated every time step Δ*t*=0.005, using Euler’s method.

## Author contributions

K.F. designed and performed experiments, analysed data and wrote the paper; H.F. developed and performed the computational simulation and wrote the paper; T.Y. designed the molecular cloning and supervised the project; M. K. and H.T. supervised the project; M.H. supervised the project and wrote the paper. All authors read and commented on the paper.

## Additional information

**Accession codes:** DNA sequence for *SpPHB*, *SpFIL* and *SpHIS4* have been submitted into the NCBI genbank database under accession codes AB938211, AB938212 and AB938214, respectively. RNA-seq data has been deposited in the NCBI sequence read archive under accession code PRJDB3436.

**How to cite this article:** Fukushima, K. *et al*. Oriented cell division shapes carnivorous pitcher leaves of *Sarracenia purpurea*. *Nat. Commun*. 6:6450 doi: 10.1038/ncomms7450 (2015).

## Supplementary Material

Supplementary Information Supplementary Figures 1-14, Supplementary Tables 1-2 and Supplementary References
