# Supplementary material for: Oriented cell division shapes carnivorous pitcher leaves of Sarracenia purpurea
Source: Nat Commun. 2015 Mar 16;6:6450. doi: 10.1038/ncomms7450 (PMC4382701; doi:10.1038/ncomms7450)
Supplement: Supplementary Information — Supplementary Figures 1-14, Supplementary Tables 1-2 and Supplementary References [file ncomms7450-s1.pdf]

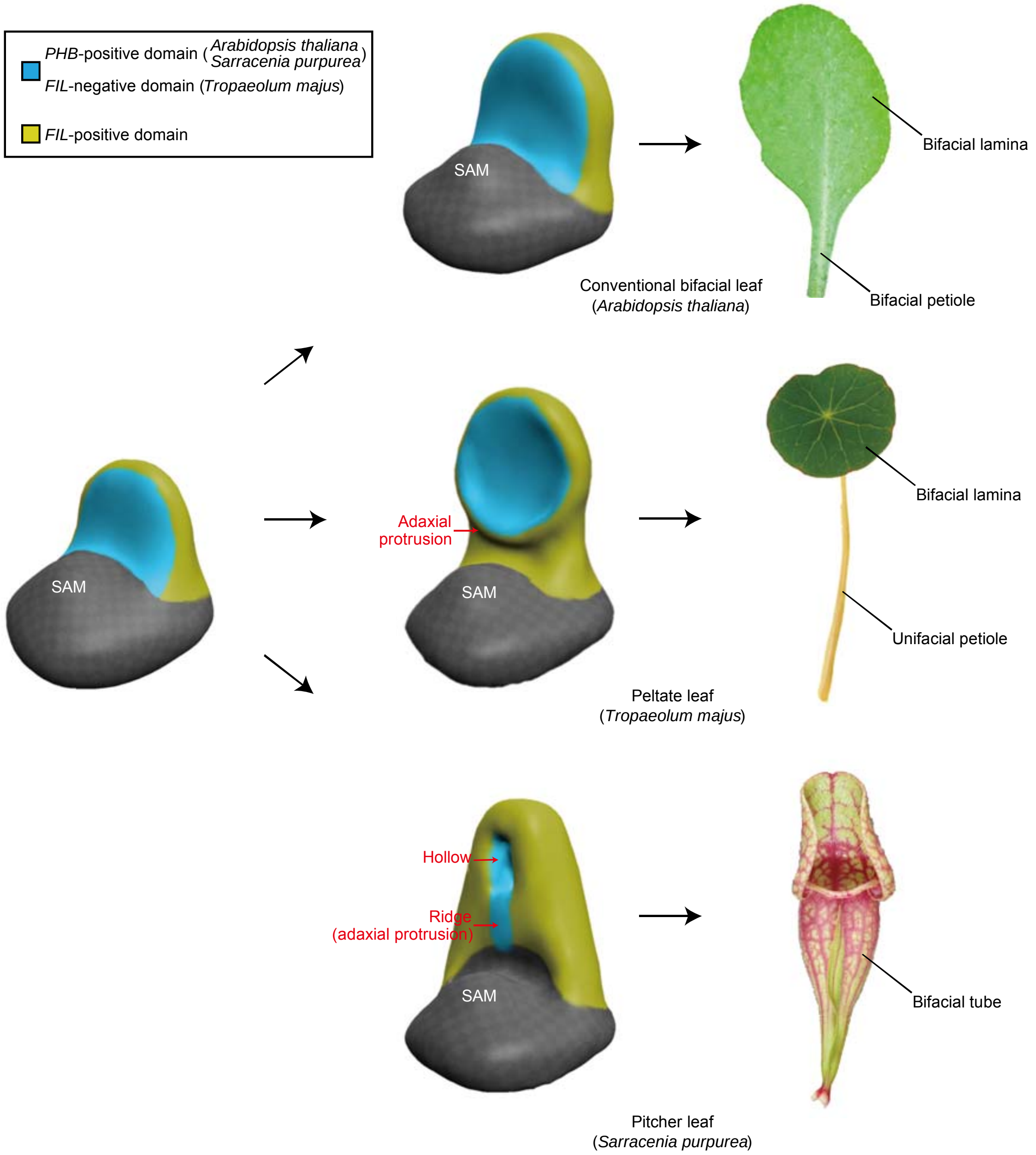

Supplementary Figure 1 | A model of leaf development through differential patterning of polarized gene expression. Expression patterns of *PHABULOSA* (*PHB*) and *FILAMENTOUS FLOWER* (*FIL*) mRNA are indicated in blue and yellow, respectively, based on studies in *Arabidopsis thaliana*[3, 4, 5, 10] and *Tropaeolum majus*[11] as well as this study on *Sarracenia purpurea*. *PHB* expression in peltate leaves has not been studied and we postulated that *PHB* would be expressed in a *FIL*-negative domain. SAM: shoot apical meristem.

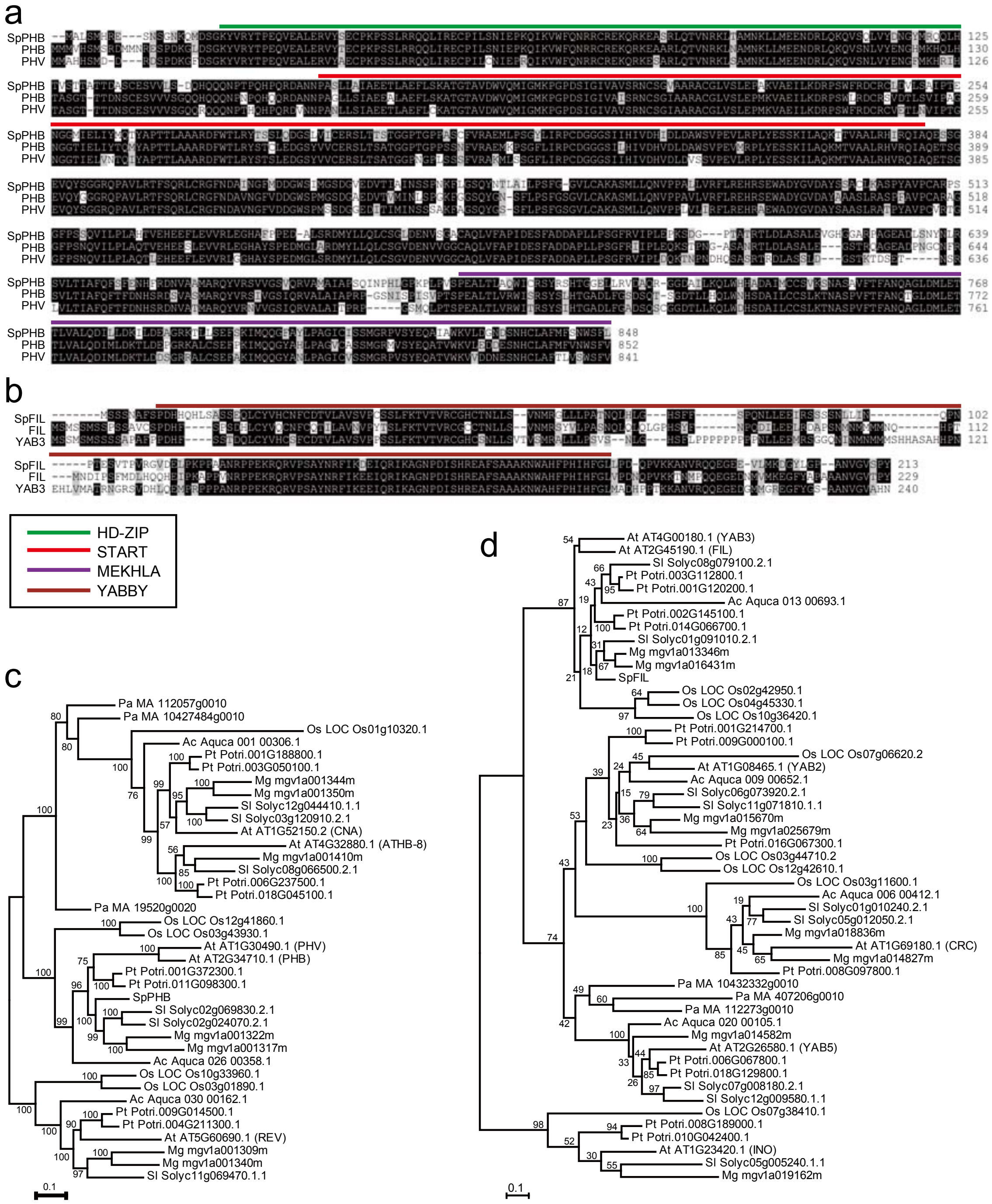

Supplementary Figure 2 | Identification of *PHABULOSA* and *FILAMENTOUS FLOWER* orthologs in *S. purpurea*. (**a,b**) Alignment of amino acid sequences of *SpPHB* (**a**) and *SpFIL* (**b**) with that of *A. thaliana* orthologs. Green, red, purple, and brown bars above the alignments indicate HD-ZIP, START, MEKHLA, and YABBY domains, respectively. (**c, d**) Unrooted maximum likelihood trees of *SpPHB* (**c**) and *SpFIL* (**d**) and their representative seed plant homologs. First two letters denote plant species and are followed by gene ID. At: *Arabidopsis thaliana*; Pt: *Populus trichocarpa*; Mg: *Mimulus guttatus*; Sl: *Solanum lycopersicum*; Ac:

Aquilegia coerulea; Os: *Oryza sativa*; Pa: *Picea abies*. Names of *A. thaliana* genes are given in parentheses. Numbers on branches are bootstrap percentages of 1000 replicates. Scale bars indicates number of amino acid substitutions per site.

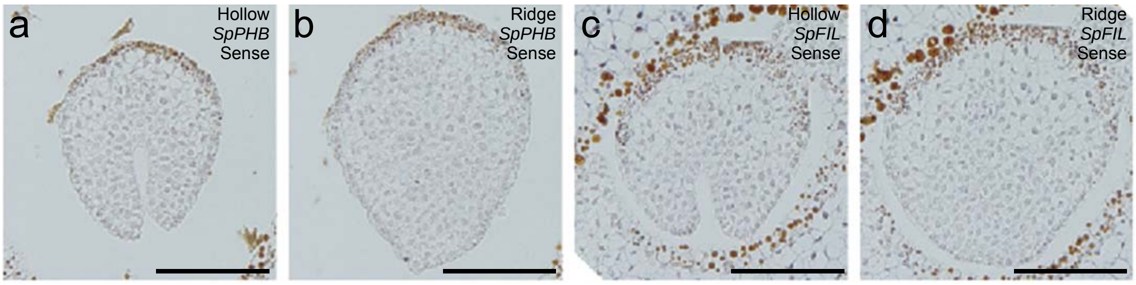

Supplementary Figure 3 | Negative control experiments of RNA *in situ* hybridization using sense probes of *SpPHB* and *SpFIL*. Transverse sections of hollow (**a**,**c**) and ridge (**b**,**d**) regions of primordia in height of ca. 210 µm (**a**,**b**) and 240 µm (**c**,**d**) are hybridized with sense probes of *SpPHB* (**a**,**b**) and *SpFIL* (**c**,**d**). The images represent three leaf primordia. Bars = 100 µm.

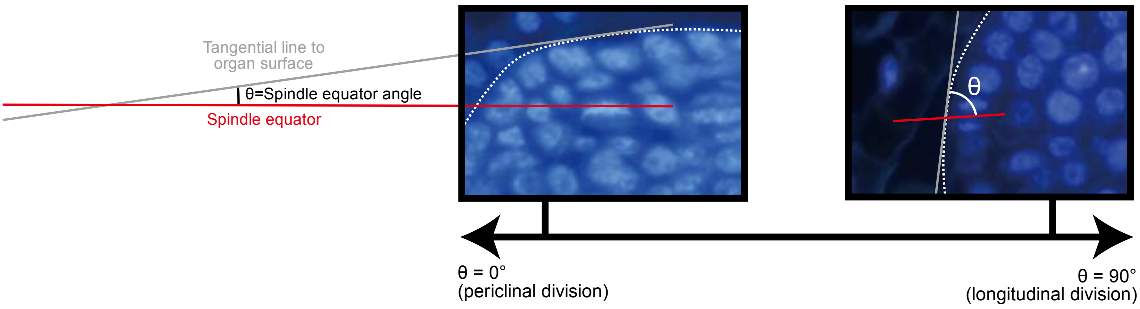

Supplementary Figure 4 | Measurement of spindle equator orientation in transverse sections. The example on the left shows measurement of the spindle equator angles during periclinal division, whereas that on the right shows measurement during longitudinal division. Dashed lines denote the organ surface.

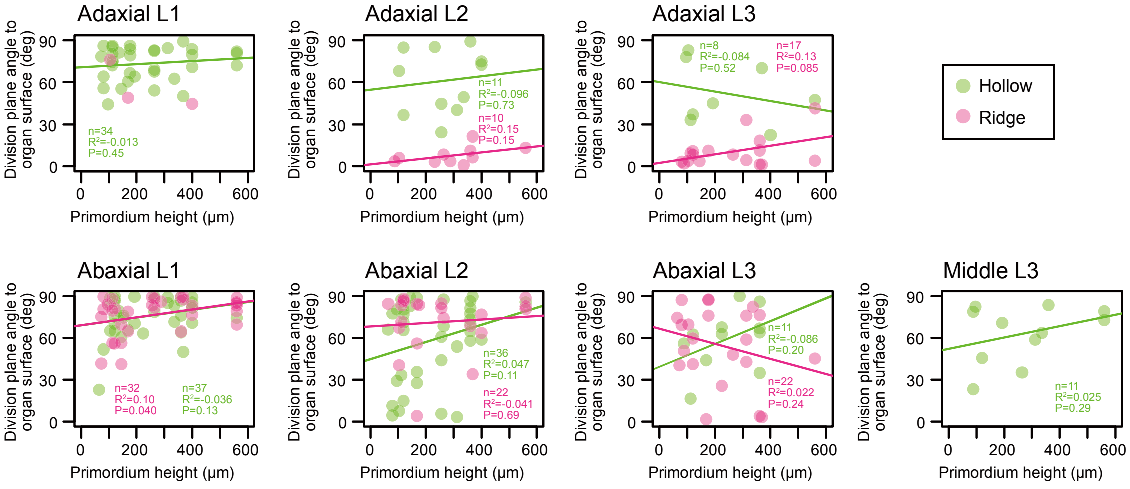

Supplementary Figure 5 | Polarity of cell division orientation in transverse sections. Division angles of different types of cells during pitcher development. Specification of cell positions is the same as in Fig. 3. Linear regression line, number of samples, adjusted R^2, and *P* value are shown in a plot. *P* values indicate a significant difference of R^2 from 0 as determined by a *F* test.

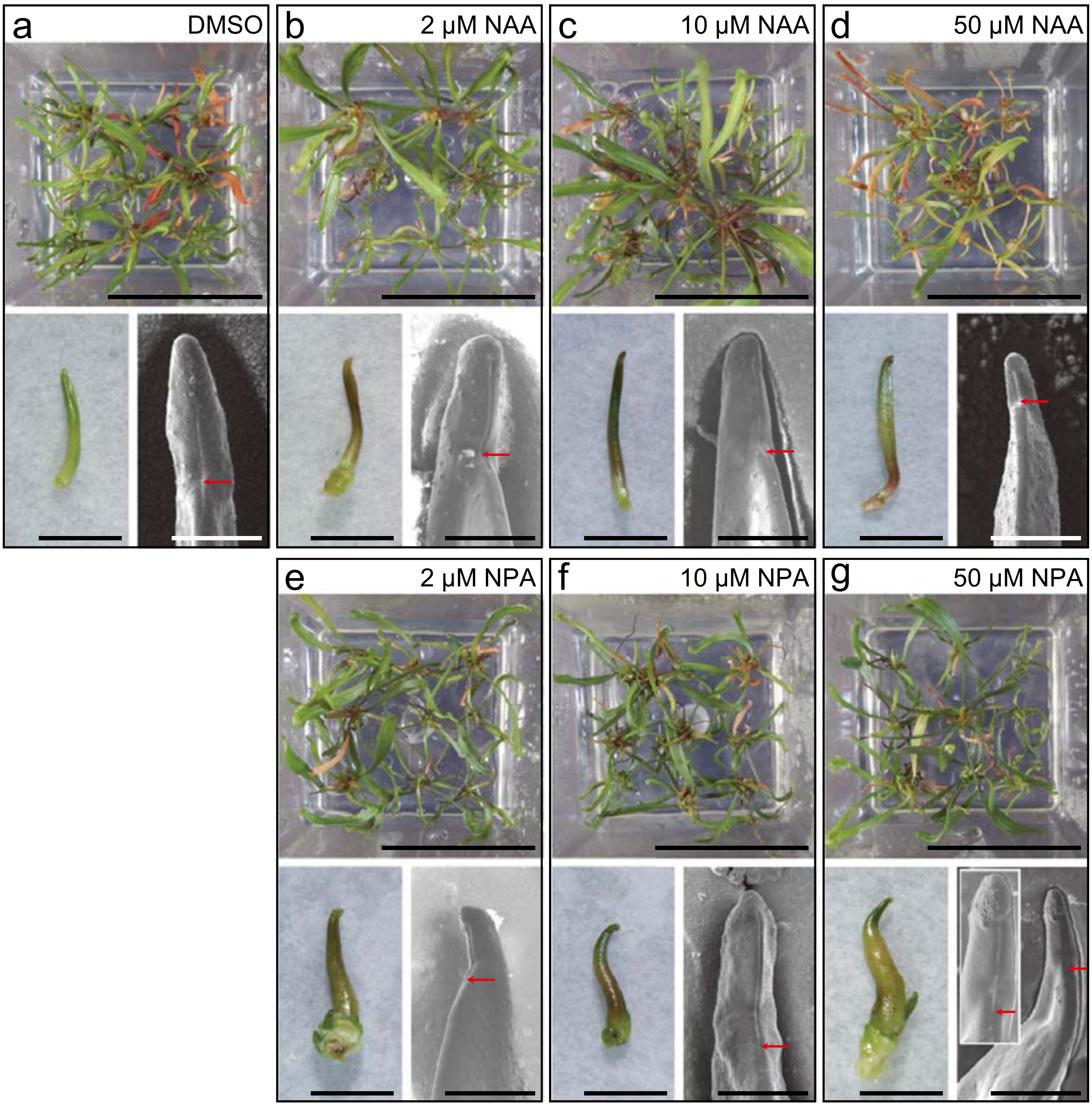

Supplementary Figure 6 | Effects of auxin addition on pitcher leaf development. Dimethyl sulfoxide (DMSO, **a**), 1-Naphthaleneacetic acid (NAA, **b-d**), and 1-N-Naphthylphthalamic acid (NPA, **e-g**) in a concentration of 2 µM (**b**,**e**), 10 µM (**c**,**f**), or 50 µM (**d**,**g**) are added to a growing medium, on which *S. purpurea* plantlets were cultivated for 4 weeks. In each panel, an upper image shows plantlets growing on the medium. A lower left image is a dissected leaf primordium in height of 5 to 10 mm. The primordium is oriented with the adaxial side right and the abaxial side left. A lower right image shows scanning electron micrograph of a leaf primordium in height of 5 to 10 mm. An inset in the lower right image of **g** is a magnified view of the distal part. Each micrograph represents at least three leaf primordia. Red arrows denote the boundary between hollow and ridge regions. Bars indicate 50 mm, 5 mm, and 500 µm for upper, lower left, and lower right images, respectively.

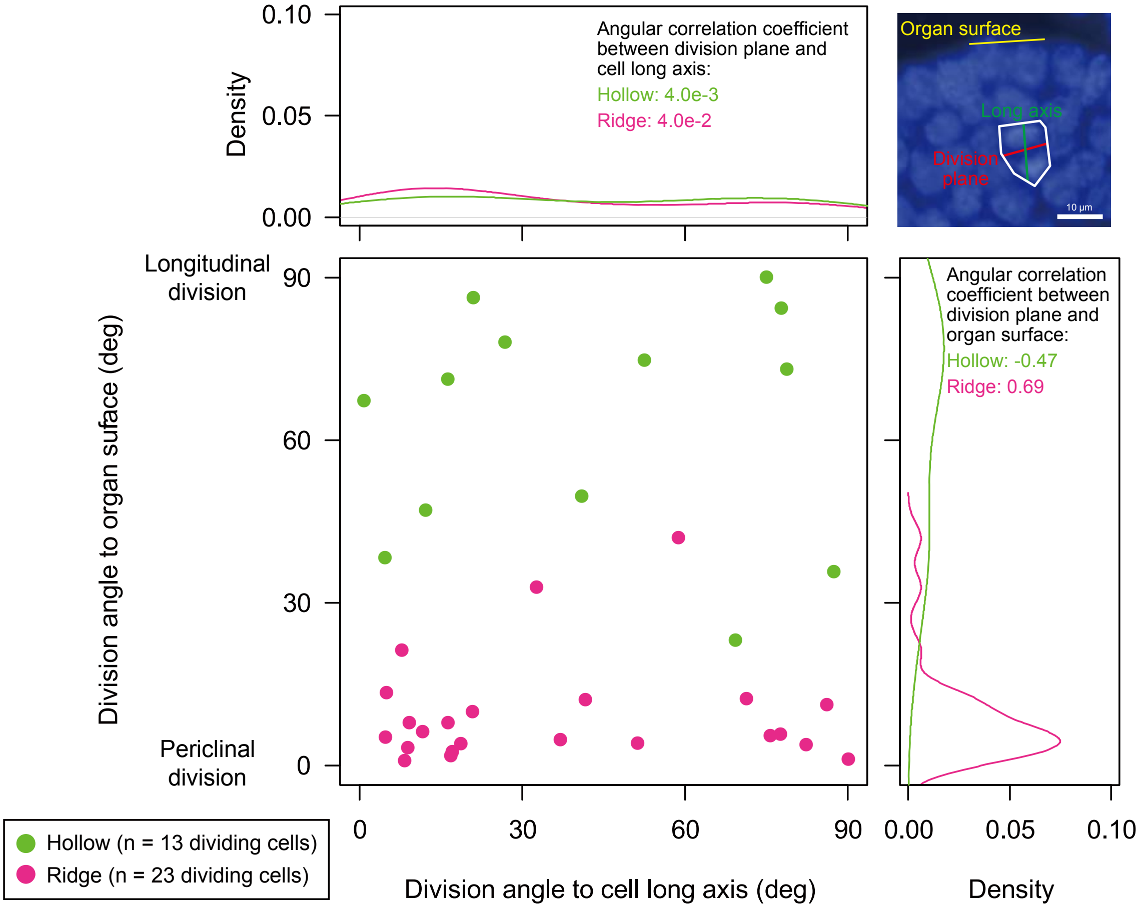

Supplementary Figure 7 | Contribution of cell shape and cell position to division orientation in adaxial L2 and L3 cells. Cell shape of dividing cells are measured in transverse sections and cell long axis is determined as the same procedure described in Model (iv) section in Methods. Upper right panel shows an example of analyzed cells. Angles formed by division plane, cell long axis, and organ surface are shown as scatter plot (bottom left). Angular correlation coefficients between division plane and organ surface are provided in density plot of bottom right panel, and those between division plane and cell long axis are shown in the plot of upper left panel.

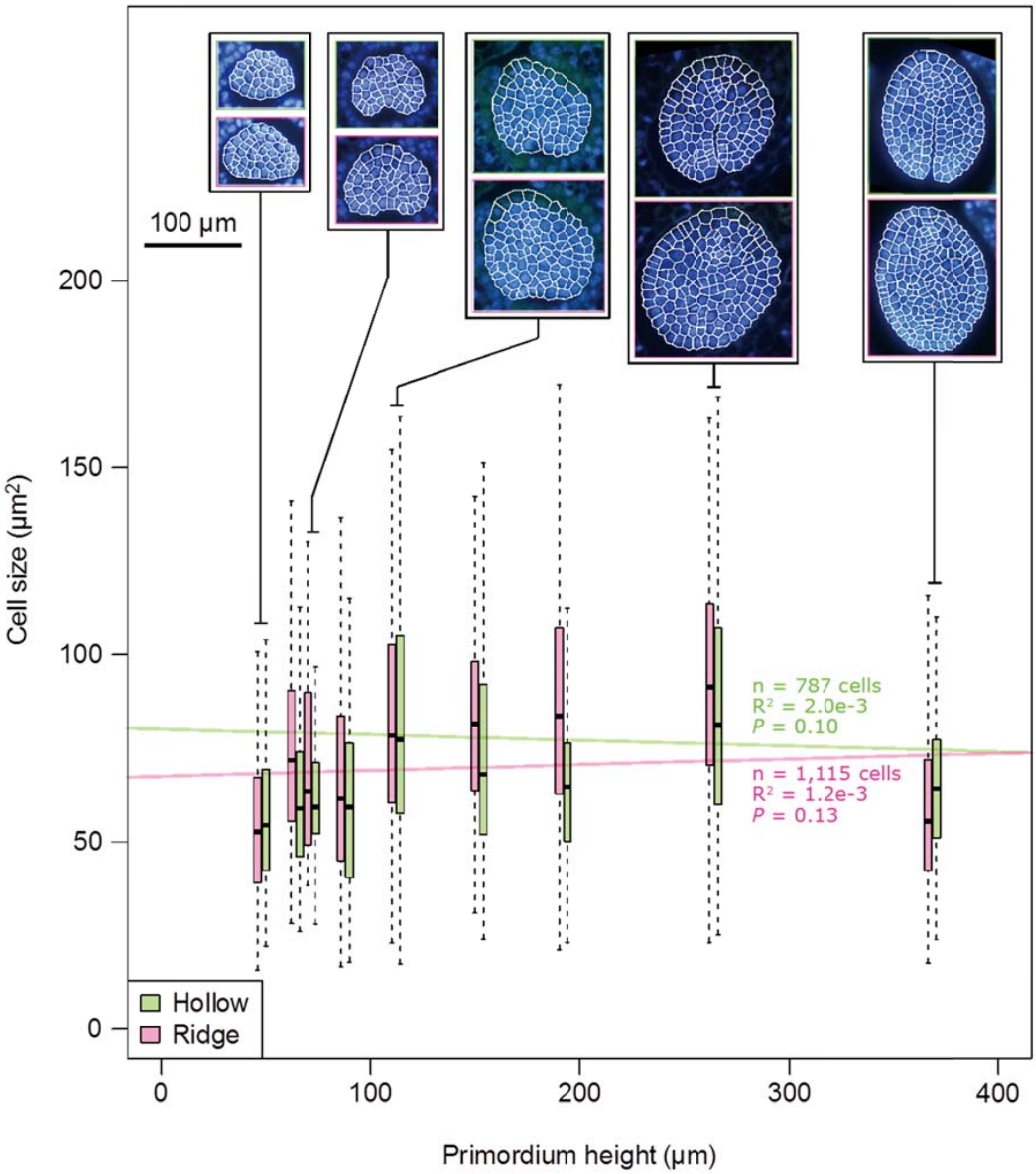

Supplementary Figure 8 | Cell size distribution in pitcher development. Cell sizes are measured in transverse sections of hollow and ridge parts and shown as a box plot. Nine primordia in different developmental stages were examined. Representative pictures of transverse sections are shown as insets. Linear regression lines, the number of measured cells (n), adjusted R^2, and *P* value are shown in the plot. *P* values indicate a significant difference of R^2 from 0 as determined by a *F* test. A bar indicates 100 µm for the microscopic images of transverse sections.

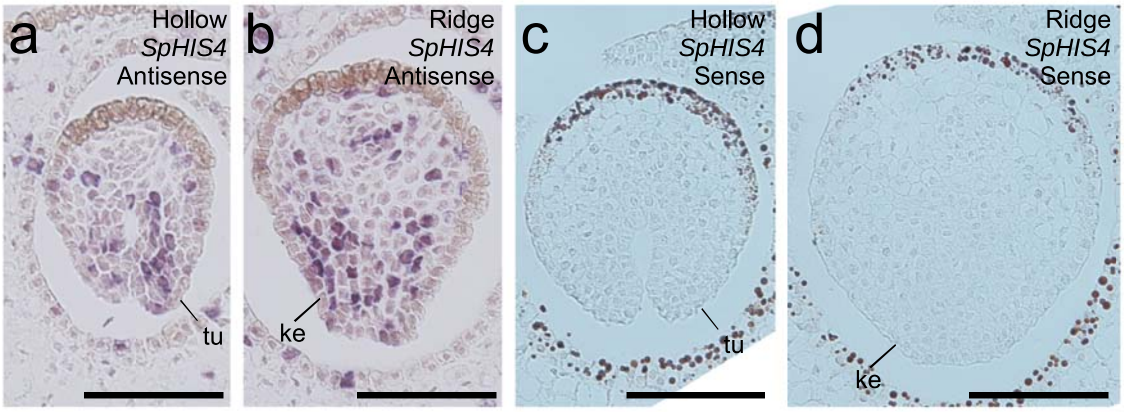

Supplementary Figure 9 | mRNA localization of *Sarracenia purpurea HistoneH4* (*SpHIS4*) in pitcher development as an indicator of cell division activity. Antisense (**a**,**b**) and sense (**c**,**d**) probes are hybridized to transverse sections of the hollow (**a,c**) and ridge (**b,d**) regions of primordia of ca. 370 µm (**a**,**b**) and 306 µm (**c**,**d**) in length. The section images of antisense and sense probe experiments represent ten and three leaf primordia, respectively. tu: tube, ke: keel. Bars = 100 µm.

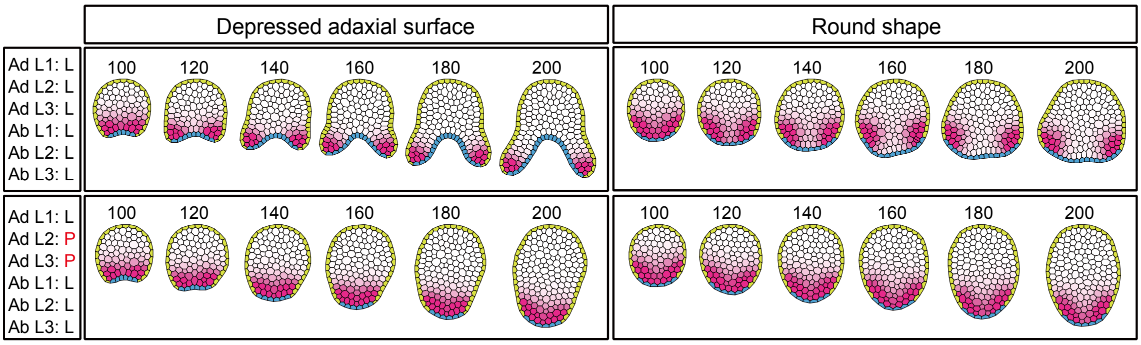

Supplementary Figure 10 | Effects of initial morphology on simulated leaf morphogenesis. Simulations are started from cell aggregates with round shape (right) and those with depressed adaxial surface (left). The preset division plane of each cell layer is indicated on the left side: L, longitudinal division; P, periclinal division.

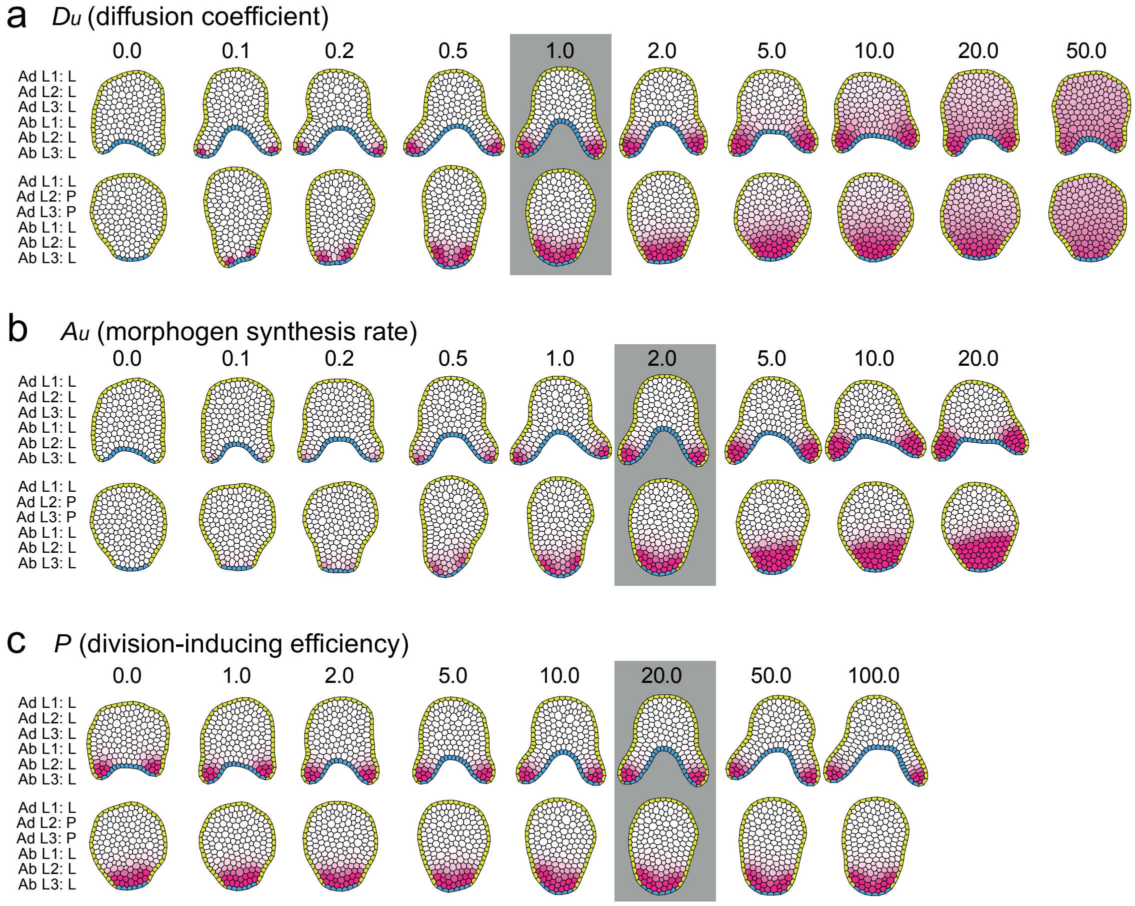

Supplementary Figure 11 | Effects of a cell division-promoting morphogen on simulated leaf morphogenesis. Diffusion coefficient (**a**), morphogen synthesis rate in adaxial-abaxial boundary cells (**b**), and a constant that affects the cell division inducing efficiency of the morphogen (**c**) are examined. The preset division plane of each cell layer is indicated on the left side: L, longitudinal division; P, periclinal division. Parameters used in Fig. 4 are shaded. Darker shade represents higher concentration.

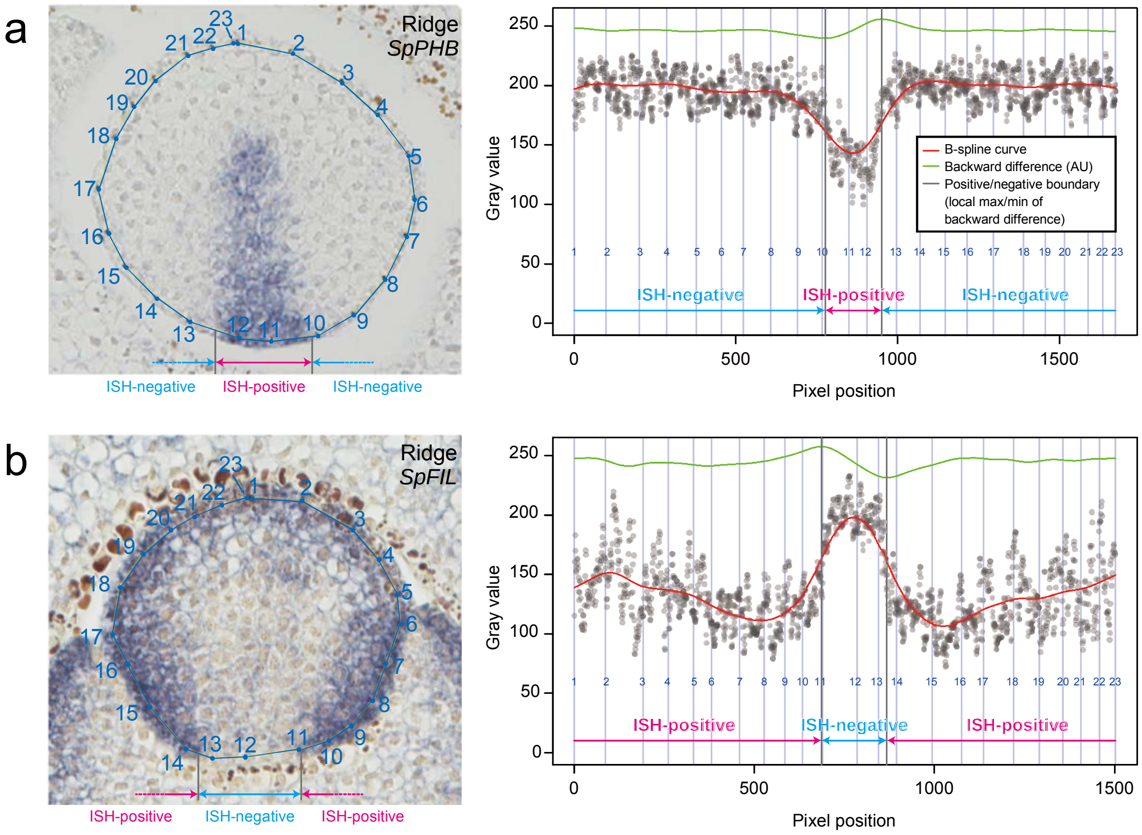

Supplementary Figure 12 | Determination of *in situ* hybridization (ISH)-positive and ISH-negative epidermal cells. Transverse sections in Fig. 2b are shown as examples of signal detection for *SpPHB* (**a**) and *SpFIL* (**b**) (left). The gray value of each pixel was measured along the blue segmented lines. Numbers indicate the position of vertices of segmented lines, and correspond to pixel positions in the graph (right). The boundary of ISH-positive and ISH-negative epidermal cells was determined by calculating the local maximum and local minimum from fitted values of the B-spline curve.

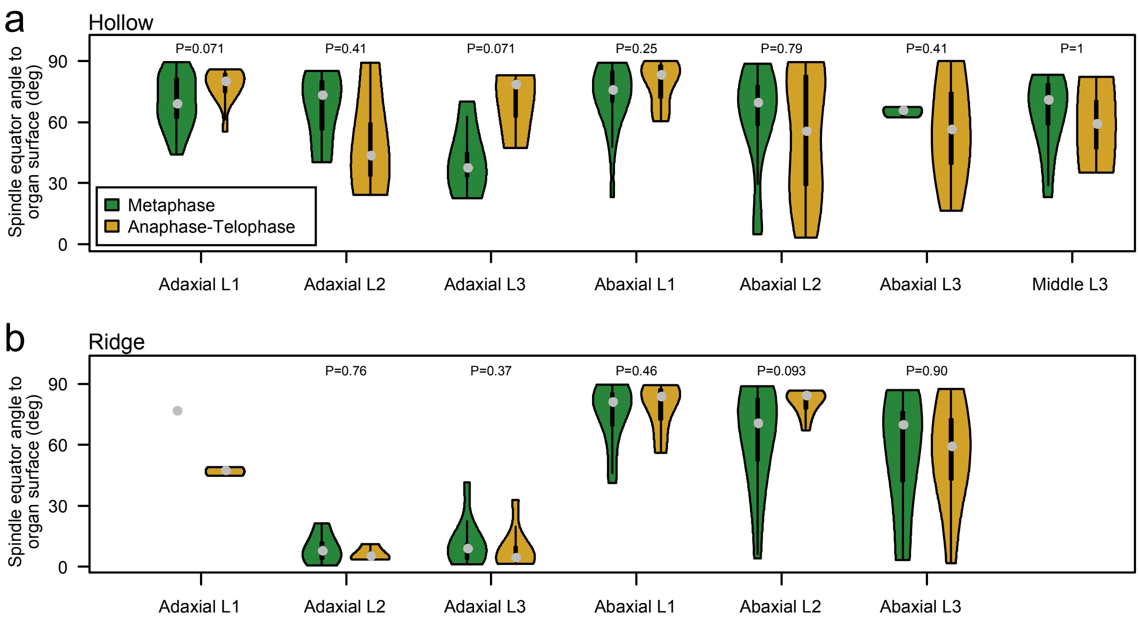

Supplementary Figure 13 | Comparison of spindle equator orientation in different cell division stages. (**a,b**) Differences of spindle equator orientation in the hollow (**a**) and ridge (**b**) regions. Angles of spindle equators are illustrated as violin plots. Gray circles indicate median values. Thick and thin lines cover ±1 and ±1.5 interquantile ranges, respectively. The vertical curve is an estimator of the density. *P* values indicate significant differences as determined by a Mann-Whitney *U* test.

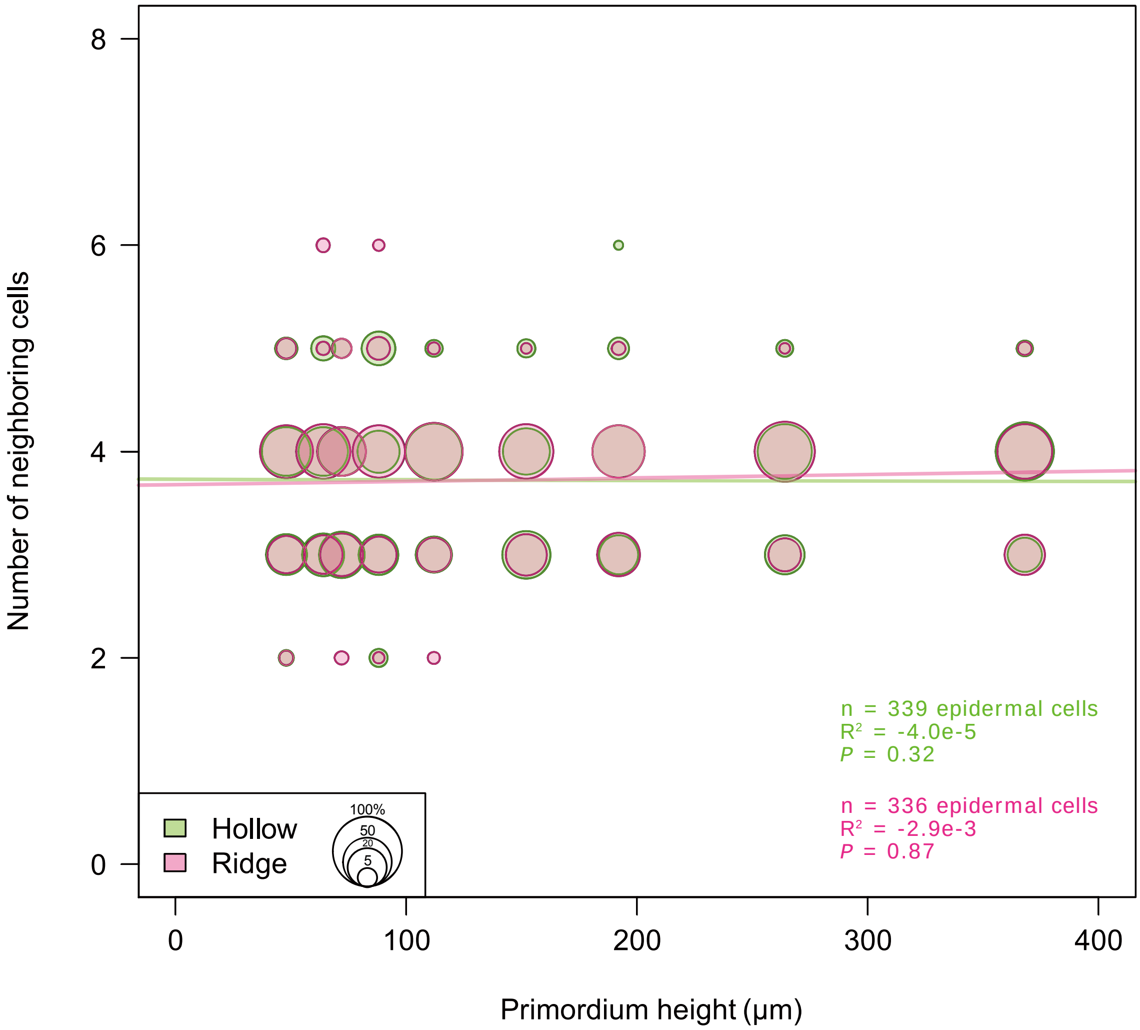

Supplementary Figure 14 | Neighboring cell numbers of epidermal cells in pitcher development. Cells that an epidermal cell contacts are counted in transverse sections and shown as a bubble plot. Data were acquired from 9 primordia with different developmental stages. Linear regression line, number of measured cells, adjusted R^2, and *P* value are shown in a plot. *P* values indicate a significant difference of R^2 from 0 as determined by a *F* test.

Supplementary Table 1 | Kolmogorov-Smirnov test for uniformity of division angles in transverse sections.

Tissue	Cell position	Number of cells examined	*P* value
Hollow region	Adaxial L1	34	7.1e-10
	Adaxial L2	11	0.18
	Adaxial L3	8	0.62
	Abaxial L1	37	7.0e-13
	Abaxial L2	36	1.4e-3
	Abaxial L3	11	0.11
	Middle L3	11	0.061
Ridge region	Adaxial L1	3	0.34
	Adaxial L2	10	1.3e-06
	Adaxial L3	17	8.2e-09
	Abaxial L1	32	5.3e-10
	Abaxial L2	22	4.0e-06
	Abaxial L3	22	0.071

Supplementary Table 2 | Oligonucleotide primers used in this study.

Gene	Primer name	Primer sequence (5' to 3')	Usage	References
SpPHB	dPHB-d7	TGCGGTACACCCCCGARCARGTNGA	degenerate PCR (1st round)	This study
	dPHB-u8	AGGAGGGCCGGTCCTTNARDATYTC	degenerate PCR (1st round)	This study
	dPHB-d5	CGAGCCCAAGCAGATCAARGTNTGGTT	degenerate PCR (nested following to dPHB-d7)	S1
	dPHB-u6	GGGCTCCAGGTTCACCARNCCRCANGC	degenerate PCR (nested following to dPHB-u8)	S1
	PHB-F1	GTCAATGGGTTTATGGATGATGGTTGG	3'-RACE	This study
	PHB-F2	ATAATGGGTAGCGATGGTGTGGAG	3'-RACE	This study
SpFIL	dYABd1	TGTTCAAGACCGTGACCGTNMGNTGYGG	degenerate PCR	This study
	dYABu4	CGGCCATGGAGAAGGCYTCNCKRTG	degenerate PCR	This study
	FIL-F1	ACTGCACCAACCTCTTGTCTGTG	3'-RACE	This study
	FIL-F2	AACATGCGTGGTCTGCTGCTTC	3'-RACE	This study
SpHIS4	dH4-d1	GGAAGGTGCTGCGGGAYAAYATHCA	degenerate PCR	S2
	dH4-u2	CCGCTTCAGGGCGTACACNACRTCCAT	degenerate PCR	S2
-	M13-21	TGTAAAACGACGGCCAGT	TA-cloning	-
	RV-17mer	CAGGAAACAGCTATGAC	TA-cloning	-

Supplementary References

1. Nakayama H, Yamaguchi T, Tsukaya H. Acquisition and diversification of cladodes: leaf-like organs in the genus *Asparagus*. *Plant Cell* 2012, 24(3): 929-940.

2. Yamaguchi T, Yano S, Tsukaya H. Genetic framework for flattened leaf blade formation in unifacial leaves of *Juncus prismatocarpus*. *Plant Cell* 2010, 22(7): 2141-2155.
